# Computational Analysis of the Micromechanical Stress Field in Undamaged and Damaged Unidirectional Fiber-Reinforced Plastics Using a Modified Principal Component Analysis

**DOI:** 10.3390/polym16213000

**Published:** 2024-10-25

**Authors:** Nicolas Rozo Lopez, Hakan Çelik, Christian Hopmann

**Affiliations:** Institute for Plastics Processing (IKV), RWTH Aachen University, Seffenter Weg 201, 52074 Aachen, Germany; hakan.celik@ikv.rwth-aachen.de (H.Ç.); publications@ikv.rwth-aachen.de (C.H.)

**Keywords:** finite element analysis, representative volume element, principal component analysis, unidirectional fiber-reinforced plastics, epoxy resin

## Abstract

This study investigated the internal stress distribution of unidirectional fiber-reinforced plastics (UD-FRP) at the micro level using principal component analysis (PCA). The composite material was simulated using a representative volume element model together with the embedded cell approach. Two fundamental quasi-static load cases, transverse and longitudinal tensile deformation, were considered. The experimental results show that mechanical failure occurred at 2.15 ± 0.06% transverse tensile strain and at 1.52 ± 0.07% longitudinal tensile strain. Furthermore, the undamaged state and a combination of matrix and interface damage, as well as fiber breakage, were simulated. From the simulations, the octahedral shear stress and octahedral normal stress were computed at the integration points of the matrix elements, constituting what is known as the octahedral stress field. A modification on the PCA to obtain mesh-independent eigenvalues is presented and was used to investigate the effects of damage events on the octahedral stress field. The results indicate that each damage mechanism had a distinct signature in the redistribution of the stress field, characterized by specific changes in the eigenvalues and orientation of the principal component (*θ*_1_). Furthermore, the PCA suggests that the accumulation of matrix damage began to be relevant at the 1% strain, while fiber breakage began at an average longitudinal strain of 0.98 ± 0.12%. Additionally, it is shown that the first principal component served as an indicator of the predominant stress state of the stress field. This investigation suggests that the PCA can provide valuable insights regarding the complex damage mechanisms of UD-FRP that may not be captured by conventional mechanical analysis.

## 1. Introduction

Fiber-reinforced plastics (FRPs) have exceptional specific mechanical properties, in which their performance depends on the individual and combined properties of the matrix and fibers, along with the fiber distribution and orientation. Thermoset matrices, characterized by high strength, stiffness, and fatigue resistance, along with carbon fibers, known for their high strength and stiffness, and glass fibers, with lower strength and stiffness but lower cost, are the still the preferred choice for high-performance composites in aerospace and automotive applications [1,2,3,4], as well as for civil engineering applications [5], even in demanding environments where carbon fibers exhibit high resistance to environmental degradation [6]. However, their exceptional specific mechanical property application in structural design is often constrained by concerns about sustainability and significant costs [1]. Therefore, a detailed understanding of the damage mechanisms of the composite is crucial to optimize the use of the material by increasing its mechanical performance [2,3,5]. Traditional experimental and analytical methods provide valuable insight for the early design with FRP. However, due to its highly anisotropic nature, classical approaches are often limited in describing the complex micromechanical damage progress that governs the overall mechanical performance of the composite [2,3,4,5,6,7]. For this reason, numerical models have become popular for addressing the open questions regarding the interaction of the various damage mechanisms, especially under combined load conditions. Finite element methods (FEMs) in combination with representative volume element (RVE) models have emerged as a well-known numerical approach in which the mean response of the reinforced material is approximated by modeling the micromechanical interaction of a small arrangement of fibers embedded within a polymer matrix [8,9,10]. Research indicates that the mechanical response of the composite depends not only on the elasto-plastic or viscoelastic properties of its individual constituents, but also on a wide range of microdamage interactions [11,12,13]. However, the accuracy of estimating mechanical properties or predicting damage initiation is highly dependent on the models used and a representative non-uniform fiber distribution [9,12]. For instance, accurate modeling of the matrix and interface damage can be achieved by stiffness degradation and traction-separation laws, respectively [14,15,16]. Moreover, the integration of RVEs with these damage laws has been found to be very useful for the evaluating of localized stress concentrations, which are critical for understanding the effects interface damage on the stiffness of FRP [9].

Even though valuable contributions have been made, most numerical analyses have been confined to the validations of the damage model assumptions by comparing the homogenized stress–strain output against traditional mechanical testing. While this is still extremely valuable, a more profound understanding of the nature of the damage interactions at the micro level, as well as their importance along different stages of the deformation, is thrown out of the analysis by considering only its homogenized mechanical response. Other works have taken a deeper look at the micro damage interaction. Zhu et al. [17] and Baris et al. [18] have numerically investigated the effects of progressive fiber breakage on the stress around broken fibers by interpreting it as a stress concentration factor. However, this approach limits the scope of the results by restricting the analysis to a region of a few fibers around a fiber break and by narrowing the interpretation of the stresses to a single quantitative factor. A numerical study of the complete stress field distribution within the matrix and its redistribution during a damage event is therefore likely to provide more detailed information than a concentration factor. This sort of numerical investigation of the matrix stress field has been performed before for unidirectional fiber-reinforced plastics (UD-FRP) under dynamic-cyclic shear deformation [19]. Here, the redistribution of the matrix stress field due to viscoelastic effects was obtained from micromechanical simulations and analyzed graphically. A similar approach was used by Koch et al. [20], where the visualization of simulated matrix strain and stress distributions was used to investigate the tensile–tensile and tensile–compression fatigue behavior of UD-FRP. For the tensile–tensile loading, the stress field is redistributed towards more tensile stresses, supporting the assumption that the tensile stress is more critical than the compressive stress in terms of crack initiation. However, the matrix stress fields, plotted as two-dimensional point clouds, were only qualitatively interpreted as mere visual variations in the point clouds, without discussing any other potential quantitative or numerical interpretation. More recently, similar diagrams have been used to investigate the effects of fiber breakage on the matrix stress field of UD-FRP under longitudinal tensile deformation [21]. In this case, the diagrams were reinterpreted using principal component analysis (PCA), which showed that a fiber break left a distinct mark on the redistribution of the matrix stress field, which can be quantified by the principal components from PCA. Although a better interpretation of the stress field was achieved, a relatively small RVE model was used. Additionally, the analysis was limited to only one damage mechanism. Since the fibers tend to have a uniform stress distribution, whereas the stresses in the matrix are non-uniform [22,23], the matrix stress field can serve as a new indicator of the nature of the damage interactions at the micro level. This procedure suggests that damage events may leave a distinct signature when the matrix stress field is redistributed. However, no experimental evidence or approach has been reported to confirm this approach, since indirect methods, such as X-ray diffraction and micro-computed tomography, have proven to be limited in measuring the internal stresses during loading [2]. Therefore, analytical and numerical analysis are the only well-established methods for investigating the stress state at the micro level. However, extracting and directly analyzing the internal stresses has proven difficult due to the large and complex data involved [19,21]. Therefore, dimensional reduction techniques must be extended and applied to more efficiently and deeply interpret the data.

PCA is a widely used dimensionality reduction technique to transform datasets of potentially correlated variables into a new set of uncorrelated variables called principal components [24,25]. Cheng et al. [26] showed the application of PCA to identify and classify impact damage in carbon-fiber-reinforced plastics by processing thermal images from eddy current pulsed thermography. However, the influence of single defects in the composite could not be completely separated. Furthermore, PCA has been used extensively to analyze data from acoustic emission sensors to characterize damages in laminated composites [27]. More recently, Sengodan [10] used PCA to predict the mechanical properties of arbitrary microstructural designs applying convolutional neural networks. However, to the best of the authors’ knowledge, PCA has not been used as a method to analyze the stress field of micromechanical simulations and differentiate between damage mechanisms. This approach can provide, however, valuable insights into complex damage behavior that may not be captured by conventional mechanical analysis. Therefore, fundamental load cases should be analyzed in the first place to evaluate the methodology.

In this paper, the microstructural damage of UD-FRP was analyzed using RVE models and PCA. Furthermore, in order to establish the PCA methodology for this type of micromechanical numerical analysis, two fundamental load cases were investigated: transverse tensile deformation, mainly dominated by the matrix, and longitudinal tensile deformation, mainly dominated by the fiber. An undamaged state, as well as damage events modeled as stiffness degradation laws for the matrix and interface, alongside fiber breakage, were simulated. The stress field within the RVE matrix was obtained from the stress values of each integration point, and PCA was used to transfer the stress field to an eigenvalue problem. The eigenvectors and eigenvalues obtained were then analyzed and correlated with the modeled damage mechanisms.

## 2. Materials and Methods

### 2.1. Specimen Preparation and Tensile Test

**Figure A1:**
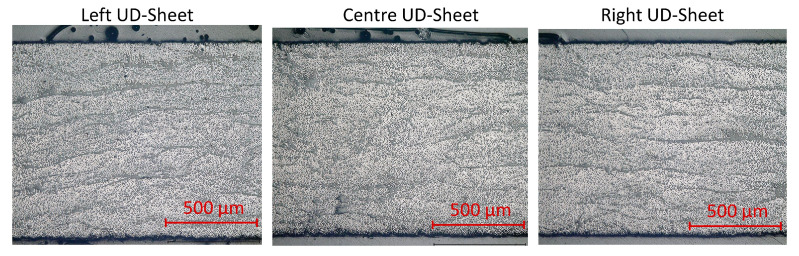
Microscopic image of the UD sheet. Samples were taken from the left, center, and right sides of the UD sheet. Average fiber volume fraction of 53 ± 1%.

Unidirectional (UD) carbon fiber sheets were manufactured using a wet-filament winding process with Araldite® LY556/HY917/DY070 epoxy resin (100:90:0.75) (Huntsman Advanced Materials, Basel, Switzerland) and Toray T700 carbon fibers (Toray, Tokyo, Japan). To minimize the influence of residual stresses, all UD sheets were cured at 80 °C for 4 h, then post-cured at 120 °C for 10 h and cooled down to room temperature. Unidirectional tensile specimens with fiber orientations of 0° (parallel to fiber) and 90° (transverse to fiber) were cut from the UD sheets according to ISO 527-5 [28]. For quality control of the tensile specimens, the microstructure and fiber volume fraction of the UD sheets were examined by light microscopy using a VHX600k microscope (KEYENCE GmbH, Neu-Isenburg, Germany). Microscopy images are shown in Appendix A (Figure A1). A self-written image processing program was used to analyze the microscopy images, estimating the fiber volume fraction by detecting and counting the fibers per unit area. The analysis showed negligible porosity in the laminate, with an average fiber volume fraction of 53 ± 1%.

Quasi-static tensile tests to failure were performed on the specimens using a Zwick Z150 universal testing machine (Zwick Roell, Ulm, Germany) equipped with a 150 kN load cell. Three repetitions were performed for each loading condition, both longitudinal tensile (parallel to the fiber) and transverse tensile (transverse to the fiber). The stress–strain curves obtained are shown in Appendix B and Appendix C (Figure A4, Figure A5, Figure A6 and Figure A7).

### 2.2. Computational Micromechanics Modeling

Volumetric RVEs were generated in ABAQUS/Standard by a previously published automatic generator [29]. Eight-node brick elements with reduced integration (C3D8R) were used for all the elements. In addition, the center node of the RVE was considered fixed ($u_{x}=u_{y}=u_{z}=0$), while displacement boundary conditions were applied to the corresponding surface nodes. For each RVE, the fiber volume fraction was calculated directly from the volume of the elements. For all simulations, an average fiber volume fraction of 50.1 ± 0.2% was obtained, which was comparable to that of the experimental specimens. The fibers were modeled as transversal isotropic linear elastic with fiber diameters of 6.9 µm. Conversely, for the deformation analyzed (quasi-static tensile loading with a maximum strain of 2%), the epoxy matrix could be assumed to behave as isotropic linear elastic solid [29]. Non-linear deformation effects were therefore not considered. The RVEs were based on Toray T700 carbon fibers with an Araldite^®^ LY556/H917 epoxy matrix. The mechanical properties are provided in Table 1.

In this study, two fundamental load cases, transverse tensile deformation and longitudinal tensile deformation along the fiber direction, were investigated. Thus, for transverse tensile deformation, the matrix is the main mechanical contributor and dominates the mechanical failure of the composite (matrix damage), while for longitudinal tensile deformation, the fibers are the main mechanical contributor and drive the failure response mainly by fiber breakage. For each loading condition, a displacement equivalent to 2% macroscopic strain was applied. Two different simulation studies were performed to investigate the mesh independence of the PCA implementation and the micromechanical stress field of UD-FRP, respectively. For each simulation study, RVE models with specific fiber arrangements and mesh configurations were used. Further details of the studies and RVEs are provided in the following sections.

#### 2.2.1. RVE for the Modified Mesh-Independent PCA Study

**Figure A2:**
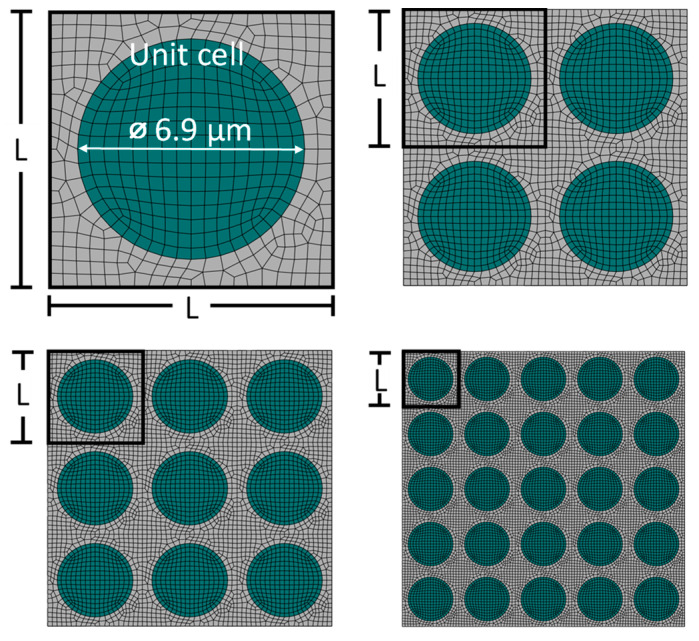
RVE with regular square pattern. Unit cell: L = 8.625 µm, thickness of 2.0 µm, and fiber diameter of 6.9 µm.

To ensure a reliable comparability of the internal stress distribution between different RVE structures using PCA, potential effects caused by the model mesh size must be removed. Therefore, any variation in the principal component (PC) will be a sole consequence of the applied load, fiber distribution, or damage, if applicable. To investigate whether the PCs were dependent on the model’s mesh, RVEs containing 1, 4, 9, and 25 fibers arranged in a regular square pattern were generated. Furthermore, micromechanical damage was not considered. To obtain identical stress fields for all the RVEs, periodic boundary conditions (PBC) and shear coupling definitions were included by means of kinematic constraints in the boundary nodes of the RVE [29]. Given the unidirectional orientation of the fibers, five elements along the fiber direction were considered sufficient to accurately reproduce the longitudinal deformation of the material. To avoid any numerical influence due to changes in the mesh geometry or number of elements, each RVE was meshed by exactly replicating the mesh on the unit cell (RVE with 1 fiber). For instance, an RVE with four fibers consisted of four repetitions of the unit cell. Details regarding the RVEs can be found in Appendix A (Figure A2).

#### 2.2.2. RVEs with Random Fiber Arrangements for the Micromechanical Stress Field Study

Three RVEs with 25 randomly distributed fibers were used to investigate the redistribution of the micromechanical stress field due to matrix damage and fiber breaks at distinct fiber distributions. The size of the RVE was 43.125 µm × 43.125 µm × 2 µm, considering that for UD-FRP it is sufficient to reproduce the longitudinal mechanical behavior accurately [29,31]. The random sequential expansion algorithm developed by Yang et al. [32] was used to achieve a more realistic fiber distribution. Since micromechanical damage was considered, the use of PBC was not suitable. Instead, an embedded cell approach (ECA) was integrated with the RVE to obtain a numerically valid mean solution for the non-periodic domain [33,34]. To avoid stress discontinuities at the interface with the RVE, a size of 150 µm × 150 µm × 2 µm was selected for the ECA. In addition, the ECA was assumed to be transversely isotropic linear elastic with mechanical properties defined by the homogenized mechanical response of the associated RVE. In this case, the displacement boundary conditions were applied to the corresponding surface nodes of the ECA.

#### 2.2.3. Micromechanical Damage Modeling Approaches

**Figure 1:**
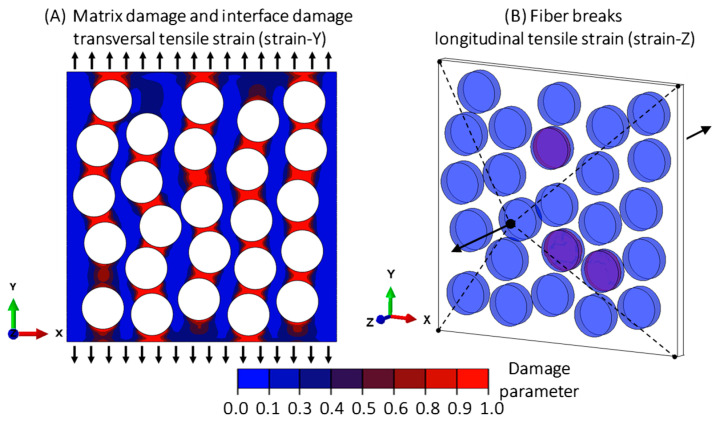
Micromechanical damage: Matrix and interface damage (**A**) and fiber breaks (**B**). For clarity, the ECA region has been omitted.

Micromechanical damage was considered in the form of stiffness degradation of the matrix elements (matrix damage) and fiber breaks. A visualization of the considered damage mechanisms is shown in Figure 1. To solve the discontinuities induced by the micro-cracks, the solver for the extended finite element method (XFEM) from ABAQUS was used. Thus, all the fibers were defined as an XFEM region, where cracks can be initiated and propagated. However, XFEM limits the use of cohesive elements with traction-separation laws, mainly due to convergence issues. Therefore, damage at the matrix–fiber interface was considered within the matrix stiffness degradation model by selecting different damage parameters for the matrix elements around the fibers. Based on Puck’s failure theory, the damage process during transverse tensile loading is mainly dominated by matrix and interface damage, while fiber breakage dominates the damage process under longitudinal tensile loading [3,13]. Therefore, fiber break mechanisms under transverse tensile loading simulations were excluded.

##### The Stiffness Degradation Model for the Matrix and Interface

Epoxy resins typically exhibit a load-dependent damage behavior, which ultimately reduces their load-bearing capacity [16]. For an epoxy matrix, this load dependency derives not only from strain-rate-dependent mechanical properties, but also from the accumulation of micro-cracks [3,35]. Stiffness degradation laws have been widely used to approximate this phenomenon while maintaining numerical stability since no finite elements need to be removed [36]. Furthermore, progressive stiffness reduction approaches have shown good agreement in modelling fiber–matrix interface debonding in unidirectional composites [37]. Although strain-rate-dependent mechanical properties were not considered in this study, matrix damage was included as a stiffness degradation law that distinguishes between matrix damage and interface damage. Both numerical and experimental investigations of epoxy matrices show that plastic deformation primarily occurs under uniaxial compression and pure shear loading conditions [31,35]. Since these load cases are not within the scope of this study, plasticity was not considered. Assuming that stiffness degradation is driven by work accumulation [38], a continuum damage model was integrated into the linear elastic model using a user material subroutine (UMAT). Thus, the damage variable D indicates the damage state (D=0 is undamaged, and D=1 is fully damaged). The effective stress $\sigma^{\prime }$ is defined as

(1) $$\sigma^{\prime }=\left(1-D\right)\sigma$$

where σ is the undamaged stress tensor. In this study, damage is distinguished between matrix damage and interface damage by uncoupling the behavior between the normal and shear stress components [31]. This is achieved by including two independent damage variables, $D_{M}$ and $D_{Int}$. Here, $D_{M}$ describes the degradation of the matrix, while $D_{Int}$ accounts for the shear stiffness degradation of the interface. By introducing two independent damage variables in the form of orthotropic Hooke’s law, the elastic modulus of the matrix elements (E) and shear modulus of the interface elements (G) are independently degraded as follows:

(2) $$E_{d}=\left(1-D_{M}\right)E$$

(3) $$G_{d}=\left(1-D_{Int}\right)G$$

where $E_{d}$ and $G_{d}$ are the resultant degraded moduli for the matrix and interface, respectively. We assume that each damage increment is proportional to the amount of dissipated energy [38], given by

(4) $$dD_{M}=A\omega \left(D_{M}\right){\left|\sigma \right|}^{n}d\sigma$$

(5) $$dD_{Int}=A\omega \left(D_{Int}\right){\left|\tau \right|}^{n}d\tau$$

where dσ and dτ are the tensile and shear stress increments, respectively. A and n are material parameters, and $\omega \left(D\right)$ is the corresponded piecewise quadratic function defined as

(6) $$\omega \left(D\right)=\left\{\;\begin{array}{c} b+a_{I}{\left(D-D_{I})\right.}^{2}\;\;for\;D\leq \;D_{I}\\ \;\;\;\;\;\;\;\;\;b\;\;\;\;\;\;\;\;\;\;\;\;for\;D_{I}<D\leq \;D_{II}\\ b+a_{II}{\left(D-D_{II})\right.}^{2}\;for\;D>D_{II} \end{array}\right.$$

where b, $a_{I}$, $a_{II}$, $D_{I}$, and $D_{II}$ are material parameters. Hohe et al. [38] have empirically validated this piecewise quadratic function and demonstrated its effectiveness in controlling the damage parameter for both tensile and dynamic-cyclic deformations. Table 2 summarizes the damage parameters used. To avoid numerical singularities, $D_{M}$ and $D_{Int}$ are limited to a maximum value of 0.95. The damage variables are updated for each time step as follows:

(7) $$D_{t+1}=D_{t}+dD$$

##### Probabilistic Modeling of Fiber Breaks

For the longitudinal tensile simulations, fiber damage is modeled as the probability of failure $P\left(\sigma \right)$, i.e., fiber break, according to a 3-parameter Weibull distribution:

(8) $$P\left(\sigma \right)=1-exp\left[-{\left(\frac{L}{L_{0}}\right)}^{\gamma }{\left(\frac{\sigma }{\sigma_{0}}\right)}^{m}\right]$$

where L denotes the fiber length; $L_{0}$ is a gauge length; and m, $\sigma_{0}$, and γ are the Weibull parameters. Table 3 summarizes the used Weibull parameters. According to the weak link theory, the fibers can be considered independent, each with their own strength distribution [39]. Thus, the tensile strength of each fiber is assigned by inverting Equation (8) and randomly assigning a probability strength value from the distribution.

### 2.3. Application of Principal Component Analysis for the Matrix Stress Field

#### 2.3.1. Octahedral Stress Field Within the Matrix

An equivalent stress is often used to facilitate the comparison of mechanical behavior under different stress conditions. For epoxy materials, it has been shown that a multiaxial failure stress can be correlated by a combination of the hydrostatic and pure shear stresses [41,42]. Therefore, in this analysis, the matrix stress field was divided into the octahedral shear stress $\left(\tau_{h}\right)$ and the hydrostatic stress $\left(\sigma_{h}\right)$. Here, the pure shear output is obtained as follows:

(9) $$\tau_{h}=\frac{1}{3}\sqrt{{\left(\sigma_{1}-\sigma_{2}\right)}^{2}+{\left(\sigma_{2}-\sigma_{3}\right)}^{2}+{\left(\sigma_{3}-\sigma_{1}\right)}^{2}}$$

where $\sigma_{1}$, $\sigma_{2}$, and $\sigma_{3}$ correspond to the principal stresses. On the other hand, the hydrostatic contribution, or octahedral normal stress, is obtained by

(10) $$\sigma_{h}=\frac{1}{3}\left(\sigma_{1}+\sigma_{2}+\sigma_{3}\right)$$

**Figure 2:**
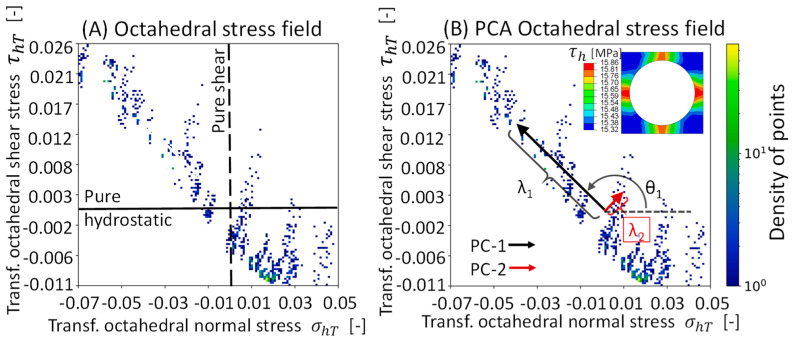
(**A**) Octahedral stress field for a unit cell under a longitudinal strain of 2% (Strain-Z). (**B**) PCA of the transformed octahedral stress field. For clarity, the fiber elements are hidden.

The octahedral stress field $\left(\sigma_{h},\;\tau_{h}\right)$ can be extracted from each integration point in the RVE matrix at the desired time increment. Figure 2A shows the octahedral stress field for the undamaged unit cell case after applying a longitudinal strain of 2%, i.e., at the last time increment. It can be observed that although an external longitudinal strain is applied, a complex stress field is formed, i.e., combination shear, tension, and compression stresses. This complex stress field can be divided into four regions, where even pure shear and pure hydrostatic stress conditions can be obtained. This is illustrated in Figure 2A. Direct observation allows for acquiring information regarding the shape and orientation of the stress distribution, its point density, and the location of the mean stresses. However, the stress field is redistributed each time a damage event occurs, adding complexity to the data interpretation.

#### 2.3.2. Implementation of Principal Component Analysis (PCA)

A Python script was implemented to compute the principal components (PCs) of the octahedral stress field. Thus, the directions in which the stress field has the most variance are described by the first and second principal components, PC-1 and PC-2, respectively. Nevertheless, PC-1 represents the direction of maximum stress variance, or the greatest amount of stress variability. To conduct the PCA, eigenvalue decomposition of the covariance matrix (Σ) is used:

(11) ΣV=λV

(12) $$\left(\begin{array}{cc} Var\left(\sigma_{h}\right) & Cov\left(\sigma_{h},\tau_{h}\right)\\ Cov\left(\tau_{h},\sigma_{h}\right) & Var\left(\tau_{h}\right) \end{array}\right)\left(\begin{array}{c} V_{1}\\ V_{2} \end{array}\right)=\left(\begin{array}{c} \lambda_{1}\\ \lambda_{2} \end{array}\right)\left(\begin{array}{c} V_{1}\\ V_{2} \end{array}\right)\;$$

where V is the orthogonal matrix of eigenvectors ($V_{1},\;V_{2})$ and λ is the diagonal matrix of eigenvalues ($\lambda_{1},{\;\lambda }_{2})$. Each PC can be decomposed into its eigenvector and eigenvalue. PC-1 $=\lambda_{1}V_{1}$ and PC-2 $=\lambda_{2}V_{2}$. To perform PCA consistently, the octahedral stress field must be transformed by centering it on the octahedral mean stresses and normalizing each stress component by its standard deviation [24]. Thus, the transformed octahedral stresses are obtained, $\sigma_{hT}$ and $\tau_{hT}$, respectively. These transformed stresses and the PC can be observed in Figure 2B for the undamaged unit cell case under 2% of longitudinal strain. In this paper, the orientation of the PC is indicated by the counterclockwise angle between the eigenvector and the octahedral normal stress axis. Therefore, the orientation of the PC-1, denoted as *θ*_1_, serves as an indicator of the predominant stress state composition within the octahedral stress field. Here, a *θ*_1_ value of 0° or 180° indicates a predominant hydrostatic stress state. Consequently, a *θ*_1_ value of 90° or 270° corresponds to a predominant shear stress state. By definition, the principal components are orthogonal, so only the orientation of PC-1 is reported.

## 3. Results and Discussion

### 3.1. Development of a Modified Mesh-Independent PCA

**Figure A3:**
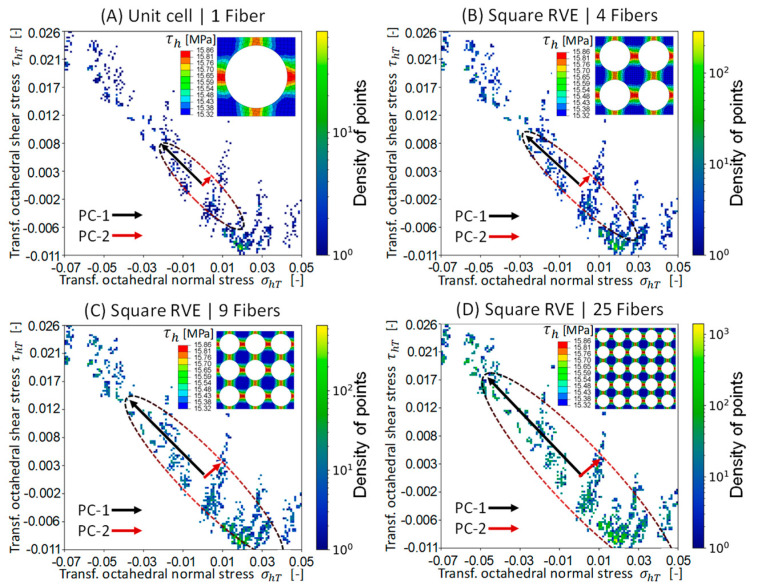
Octahedral shear stress field for the RVE models with a square fiber arrangement under longitudinal strain of 2%. (**A**) Unit cell, (**B**) RVE with four fibers, (**C**) RVE with nine fibers, and (**D**) RVE with four twenty-five.

The effect of the RVE mesh on the PCA values was investigated using the models described in Section 2.2.1. By replicating the unit cell and imposing a strictly regular fiber arrangement with PBC, all the analyzed RVEs possessed structural equivalence. The results of the octahedral shear stress for all longitudinal strain simulations can be seen in Appendix A (Figure A3). The uniformity of the stress magnitude across the RVEs confirms the correctness of the mesh replication and the implementation of PBC. As expected, the density of points within the stress field increased with the size of the RVE, since more integration points share the exact same stress state due to the exactly replicated mesh. In addition, the size of RVEs can be reinterpreted as the number of integration points within the matrix. By extracting the octahedral stress field $\left(\sigma_{h},\;\tau_{h}\right)$ from all four RVE modela and applying the conventional PCA, it is clear that the eigenvalues $\left(\lambda_{1},{\;\lambda }_{2}\right)$ show a mesh-dependent behavior, as the eigenvalues increased logarithmically with the number of integration points. As expected, *θ*_1_ remained unchanged, suggesting a correlation with the stress state. Table 4 shows the results of the PCA. For the longitudinal tensile simulation, *θ*_1_ indicates a large stress variation towards low positive hydrostatic stress accompanied by high octahedral shear stresses. While for the transverse tensile simulations, *θ*_1_ indicates a predominant stress variation towards high hydrostatic and high octahedral shear stresses.

Further analysis of the PCA shows that the covariance matrix (Σ), especially the variance entries, scaled with the number of integration points, although the stress state was the same. In order to obtain a mesh-independent PCA, the eigenvalues were scaled against the size of the octahedral stress field $\left(\sigma_{h},\;\tau_{h}\right)$ as follows:

(13) $$\left|\lambda \right|=\sqrt{\frac{\lambda^{2}}{\|\left[\sigma_{h},\;\tau_{h}\right]{\|}_{2}}}$$

where $\left|\lambda \right|$ are the modified eigenvalues and $\|\left[\sigma_{h},\;\tau_{h}\right]{\|}_{2}$ is the L2-norm of the squared matrix obtained from the octahedral stress field. The results of the modified PCA are shown in Table 4.

### 3.2. Analysis of the Micromechanical Stress Field of RVE with Random Fiber Arrangements

The analysis of the micromechanical stress field redistribution due to matrix damage and fibers breaks was performed using the mesh-independent PCA on the models described in Section 2.2.2. Each micromechanical damage mechanism was first simulated separately to analyze the effects of each damage individually. However, since the interface damage can be considered as a result of failure in the matrix, it should not be treated as an isolated damage mechanism [13,43]. Therefore, the interface bonding behavior, modeled as a progressive stiffness reduction law, was integrated into the UMAT instead of being treated as a separate subroutine. Thus, interface damage was analyzed alongside matrix damage and involved only the octahedral stress field from the elements around the fibers. For the longitudinal strain simulations, both matrix damage and fiber breaks were analyzed together. The analysis is presented by load cases, 2% transverse tensile strain (Strain-Y), and 2% longitudinal tensile strain (Strain-Z).

#### 3.2.1. Validation of the RVE Models with Random Fiber Arrangements

**Figure A4:**
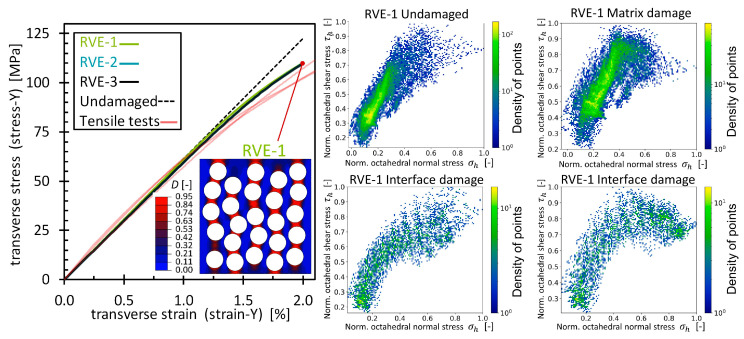
Experimental and simulated stress–strain curves and evolution of the matrix damage and the interface damage. The octahedral stress fields were obtained from the undamaged simulation and from the last simulation step. For a better visual comparison, the octahedral stresses have been normalized to the corresponding maximum value. For the sake of clarity, the ECA region has been excluded.

**Figure A5:**
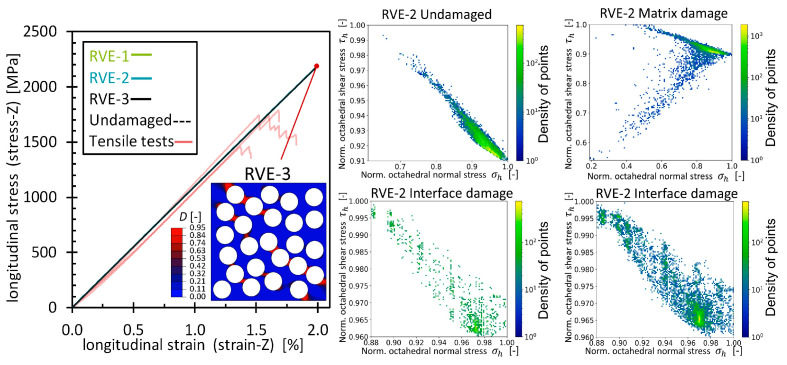
Experimental and simulated stress–strain curves and evolution of the matrix damage and the interface damage. The octahedral stress fields were obtained from the undamaged simulation and from the last simulation step. For a better visual comparison, the octahedral stresses have been normalized to the corresponding maximum value. For the sake of clarity, the ECA region has been excluded.

**Figure A6:**
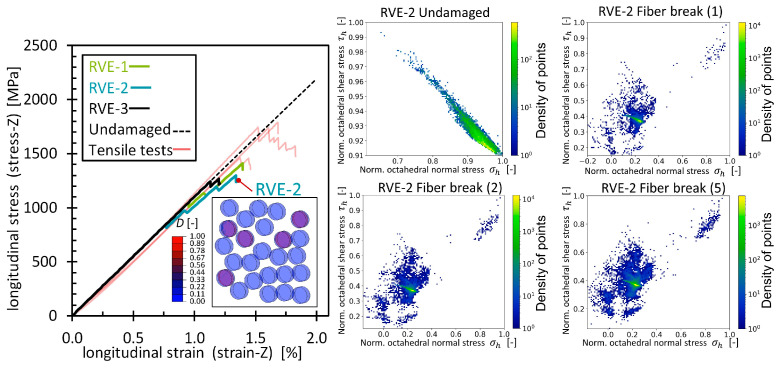
Experimental and simulated stress–strain curves and evolution of the fiber breaks. The octahedral stress fields were obtained from the undamaged simulation and from the last simulation step. For a better visual comparison, the octahedral stresses have been normalized to the corresponding maximum value. For the sake of clarity, the ECA region has been excluded from the figures.

**Figure A7:**
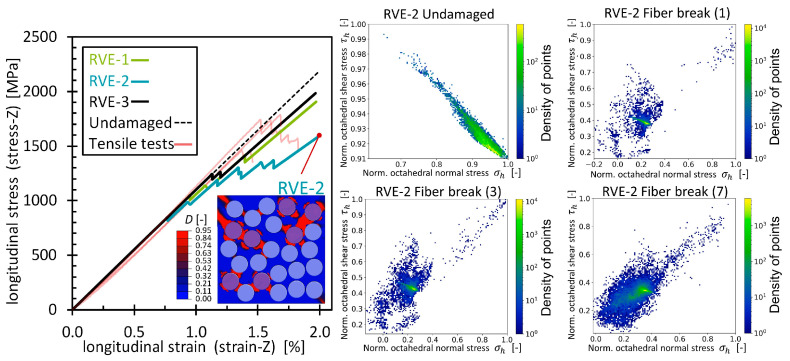
Experimental and simulated stress–strain curves and evolution of the fiber breaks together with matrix damage and the interface damage. The octahedral stress fields were obtained from the undamaged simulation and from the last simulation step. For a better visual comparison, the octahedral stresses have been normalized to the corresponding maximum value. For the sake of clarity, the ECA region has been excluded from the figures.

The simulated stress–strain curves, along with the experimental results, are shown in Appendix B and Appendix C (Figure A4, Figure A5, Figure A6 and Figure A7). The experimental results show that mechanical failure occurred at the 2.15 ± 0.06% transverse tensile strain and at the 1.52 ± 0.07% longitudinal tensile strain. Although each RVE had a different fiber distribution, a comparable mechanical response was able to be observed in all models due to the similar fiber volume fraction. However, for the longitudinal tensile simulations (Figure A5, Figure A6 and Figure A7), differences in the resulting stiffness decrease were observed across the RVEs, which can be attributed to the fiber distributions and the number of fiber breaks obtained. Furthermore, when only matrix damage was considered (Figure A5), the numerical results did not agree with the experimental observations, as sudden damage behavior was not reproduced. In this case, a small amount of matrix damage gradually accumulated without a significant effect on the RVE stiffness. In contrast, when only fiber breaks were considered (Figure A6), the numerical results partially agreed with the experimental observations. While sudden damage behavior was captured, numerical problems prevented the simulations from reaching the target strain. Nevertheless, the finding points to fiber breakage as the primary damage mechanism for this loading condition. By considering both fiber breakage and matrix damage (Figure A7), the simulations were able to achieve 2% strain. Furthermore, the RVE stiffness decrease was comparable to the experimental results, showing a sudden drop in stiffness due to a fiber break, followed by a load-carrying phase until the next fiber break event occurred, resulting in another sudden decrease in stiffness.

For the transverse tensile simulations (Figure A4), all RVEs consistently reproduced the undamaged stiffness response of the material. As significant matrix damage accumulated, a nonlinear decrease in stiffness was observed in both the experimental and simulated stress–strain curves, with the simulations slightly overestimating the mechanical response.

#### 3.2.2. Modified PCA of Transverse Tensile Strain Simulations (Matrix/Interface Damage)

**Figure 3:**
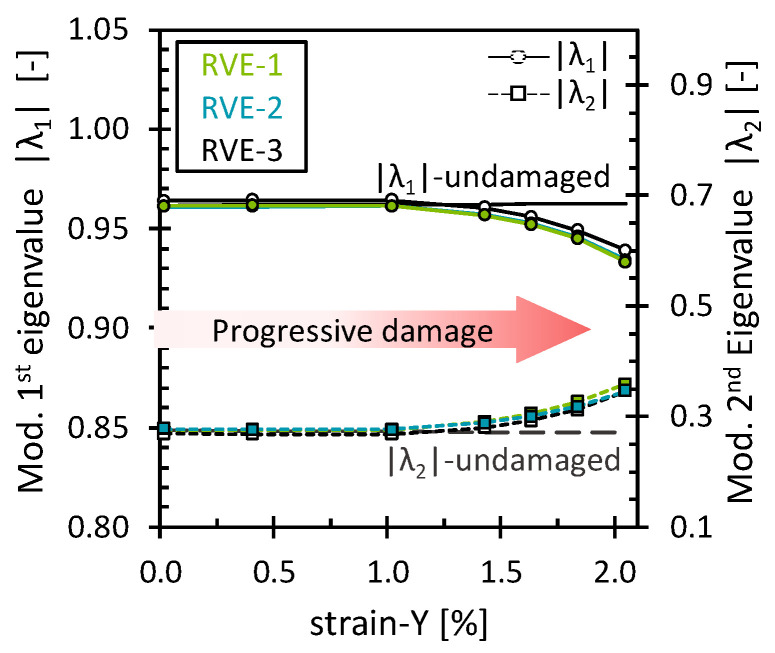
Modified eigenvalues for the matrix damage as a function of the transverse strain.

The effect of the matrix damage on the octahedral stress field was analyzed for simulations under the 2% transverse tensile strain. As mentioned above, matrix damage and interface damage were treated together. No additional damage mechanisms were considered. Since the load acted transversely to the fibers, the progressive matrix damage showed a dominating effect on the stiffness response of the RVE. The stress–strain curve and the evolution of the octahedral stress can be found in Appendix B (Figure A4). Figure 3 shows the evolution of the first and second modified eigenvalues $\left(|\lambda_{1}\left|,\;\right|\lambda_{2}|\right)$ through the deformation process. As expected, both eigenvalues remained unchanged and closer to the eigenvalues of the undamaged state as long as no critical damage accumulation occurred. Once a certain amount of damage had accumulated, as evidenced by a noticeable loss in RVE stiffness, the eigenvalues started to change gradually. Since the eigenvalues are the factor by which the eigenvector is stretched or compressed, the observed variations suggest a characteristic redistribution in which the octahedral stress field becomes progressively less oriented towards PC-1 and more oriented towards PC-2.

**Figure 4:**
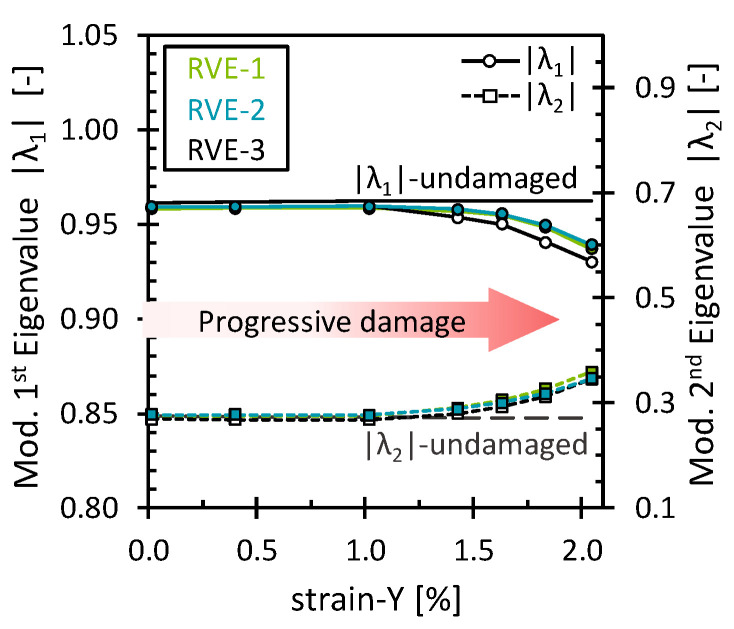
Modified eigenvalues for the interface damage as a function of the transverse strain.

**Figure 5:**
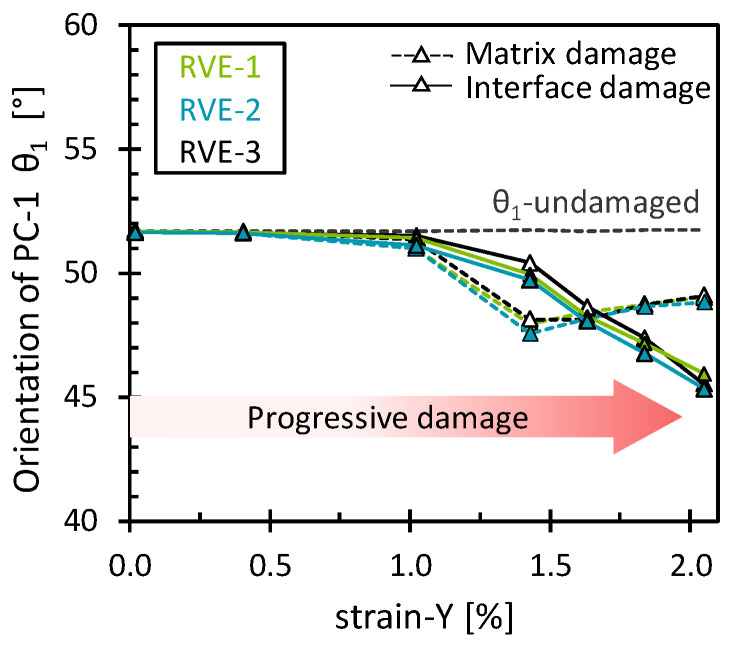
Orientation of the first eigenvector for the matrix damage and the interface damage as a function of the transverse strain.

Similar behavior was observed for the interface damage eigenvalues, which showed minimal differences compared to the matrix damage eigenvalues (Figure 4). This is presumed to result from the gradual development of both matrix and interface damage as the RVE was transversely stretched. However, this was not the case for the orientation of the first eigenvector (*θ*_1_), as it exhibited distinct patterns for each damage mechanism (Figure 5). In the case of interface damage, the stress field gradually rotated clockwise, indicating a progressive shifting of the stress field towards higher octahedral normal stresses. According to Hu et al. [41], high positive hydrostatic stress may be associated with a more brittle fracture failure of epoxy material. This suggests that interface damage is prone to a more brittle failure nature. In contrast, a more abrupt clockwise rotation was observed for matrix damage. A critical point in the reorientation of the stress field was then observed at the 1% strain, marked by a progressive counterclockwise rotation, suggesting that after a certain damage accumulation, the stress field is reoriented to a lower octahedral normal stress but with still higher octahedral shear stresses compared to the initial undamaged state. This combination of octahedral normal stress and octahedral shear stress can potentially influence the yield behavior of epoxy polymers, as suggested by Kody et al. [42]. As hypothesized, each damage mechanism left a characteristic signature in the stress field reorientation. In addition, the results obtained showed a consistent pattern across the RVEs analyzed, with slight variations due to the different fiber arrangements.

#### 3.2.3. Modified PCA of the Longitudinal Tensile Strain Simulations (Matrix/Interface Damage)

**Figure 6:**
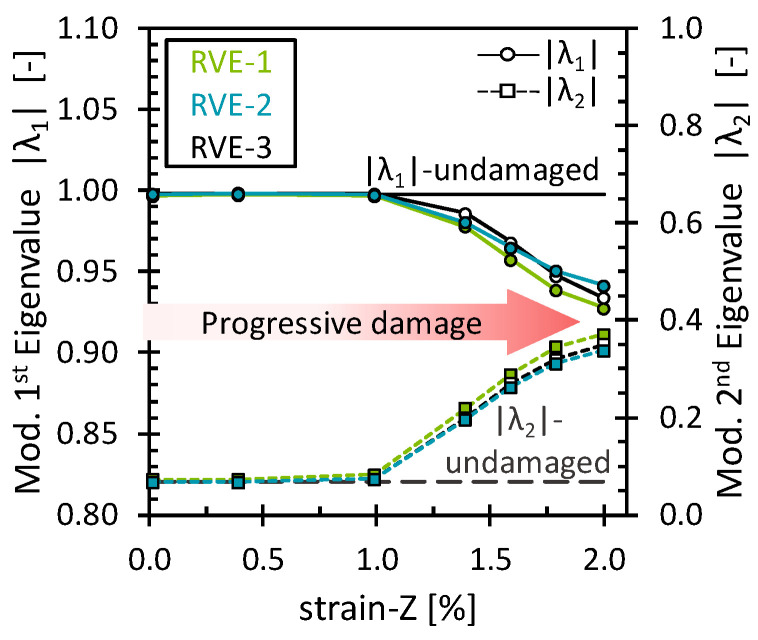
Modified eigenvalues for the matrix damage as a function of the longitudinal strain.

**Figure 7:**
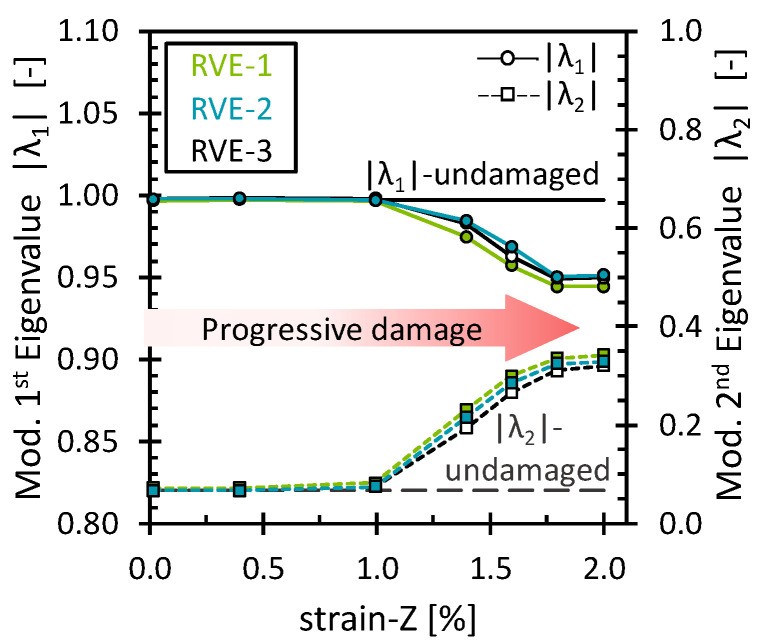
Modified eigenvalues for the interface damage as a function of the longitudinal strain.

The effect of the matrix damage on the octahedral stress field was analyzed for simulations under the 2% longitudinal tensile strain. As mentioned above, matrix damage and interface damage were treated together. Fiber breakage has not yet been considered. Since the load was applied in the direction of the fibers and no fiber damage was considered, the fibers still carried most of the load, and then the progressive matrix damage showed almost no effect on the stiffness of the RVE. The stress–strain curve and the evolution of the octahedral stress can be found in Appendix C (Figure A5). Figure 6 shows the evolution of $\left|\lambda_{1}\right|$ and $\left|\lambda_{2}\right|$ through the deformation process. As expected, both eigenvalues remained unchanged as long as no critical damage accumulation occurred. Surprisingly, the eigenvalues began to change gradually, although there was no noticeable loss in the stiffness of the RVE. Nevertheless, by observing the damage level of the matrix, it is clear that some damage accumulated while the fibers were still carrying most of the load. This shows that even though no permanent change in the mechanical performance of the RVE was observed, the eigenvalues were able to capture the damage at the matrix level, around 1% strain. Furthermore, similar to the transverse loading case, the variations of the eigenvalues suggest a similar redistribution in which the octahedral stress field becomes less oriented towards PC-1 and more oriented towards PC-2. For this load case, however, a more linear pattern and larger changes in values were observed. This can be attributed to the damaged matrix elements, where both the octahedral normal stress and the octahedral shear stress decreased due to the stiffness degradation of the elements. Therefore, the stress field reconfigured to a state where more stress points gradually obtained a low-stress value. Similar behavior was observed for the interface damage eigenvalues (Figure 7). However, an interesting difference compared to the matrix damage eigenvalues is noted for the last eigenvalues, which remained nearly constant. Upon examining the interface damage state, it was observed that all interface elements had almost reached full damage. Since no further damage could have been applied, no alterations in the scaling of the stress field occurred, reaffirming that the eigenvalues consistently capture the damage evolution.

**Figure 8:**
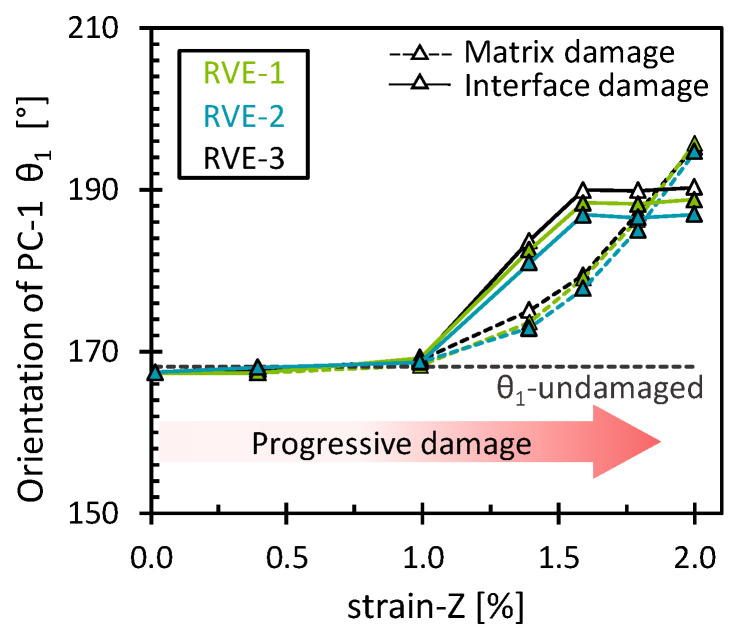
Orientation of the first eigenvector for the matrix damage and the interface damage as a function of the longitudinal strain.

In addition, *θ*_1_ showed a different pattern for each damage mechanism (Figure 8). For the matrix damage, the stress field gradually rotated counterclockwise, contrary to the behavior observed in the case of transverse tension. Thus, the stress field shifted to a predominant hydrostatic stress field (*θ*_1_ = 180°) and subsequently surpassed it due to the accumulation of low octahedral shear stress from the damaged elements. For interface damage, an abrupt counterclockwise reorientation of the stress field was observed, with a posterior stagnation suggesting the attainment of a full damage state. At the stagnation, it is interesting to note that *θ*_1_, with a mean value of 188.4° ± 1.3°, implied that the interface stress field was closely aligned with a predominant hydrostatic stress field. This suggests, as in the previous analysis, that interface damage is prone to a more brittle failure nature. As before, the results obtained show a consistent pattern across the RVEs analyzed, with slight variations due to the different fiber arrangements.

#### 3.2.4. Modified PCA of the Longitudinal Tensile Strain Simulations (Fiber Breaks)

**Figure 9:**
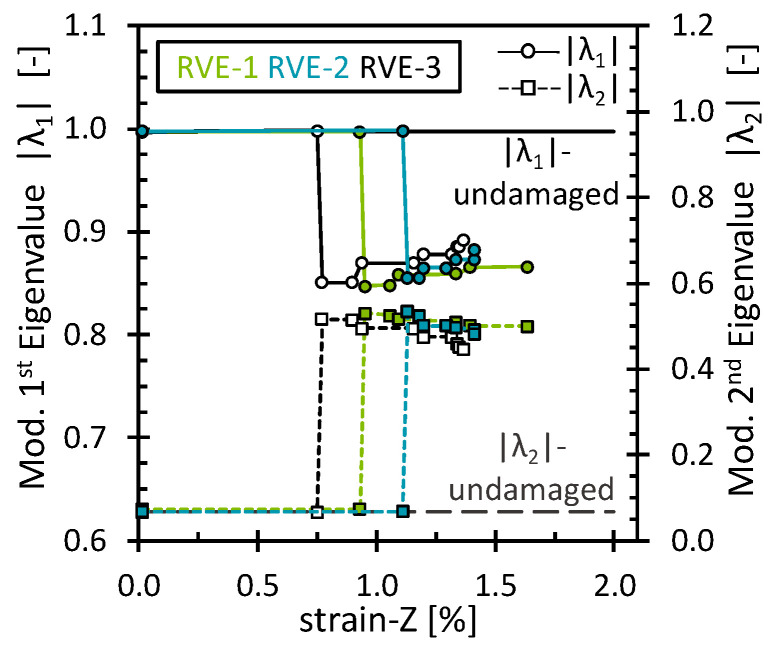
Modified eigenvalues for fiber break damage as a function of the longitudinal strain.

**Figure 10:**
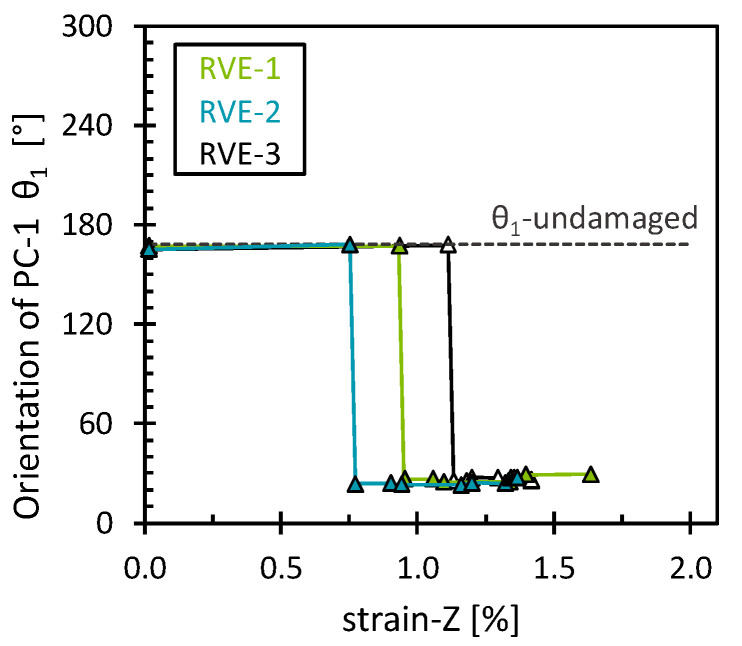
Orientation of the first eigenvector for the fiber break damage and the interface damage as a function of the longitudinal strain.

The effect of the fiber breaks on the octahedral stress field was analyzed for simulations under the 2% longitudinal tensile strain. No other damage mechanisms were considered. Since the load was applied in the direction of the fibers, and fiber breakage can occur, the overall stiffness of the RVE was drastically affected as more fibers were damaged. Therefore, the matrix, which had a lower stiffness, must transfer the load to the undamaged fibers. The stress–strain curve and the evolution of the octahedral stress can be found in Appendix C (Figure A6). Since the convergence conditions were not met due to the high degree of damage, the simulations were aborted before the final strain of the 2% strain was reached. Figure 9 shows the evolution of $\left|\lambda_{1}\right|$ and $\left|\lambda_{2}\right|$ through the deformation process. As expected, both eigenvalues remained unchanged as long as no fiber breaks occurred. However, as soon as the first break occurred, a first abrupt change was observed. Moreover, for each fiber break, the eigenvalues showed significant changes followed by steady behavior as no new fiber breaks occurred. This confirms that the stress field in the matrix was drastically affected by the first fiber break and reoriented by each subsequent break. Furthermore, with the first damage event, the octahedral stress field suddenly became less oriented toward PC-1 and more oriented to PC-2, indicating a spreading of the stress field over different values of octahedral normal stress and octahedral shear stress. This observation is evident in the octahedral stress field plots in Appendix C (Figure A6). First, a small cluster characterized by high octahedral normal stress and high octahedral shear stress was formed, presumably corresponding to the elements around the fiber break; second, a compression region was formed where, according to Hu et al. [41], ductile yielding may predominate since the hydrostatic stresses are zero or negative. However, with each subsequent fiber break, the eigenvalues showed a reversal in behavior, indicating that the octahedral stress field became increasingly aligned with PC-1 and PC-2. This suggests that the octahedral stresses tend to maintain the pattern established by the initial break, with each subsequent fiber break generating stress states that adhere to this initial pattern. This can also be observed in *θ*_1_ (Figure 10), where the stress field immediately rotated clockwise without much variation thereafter. For *θ*_1_, a mean value of 25.08° ± 1.77° after the first break was obtained. This implies that the stress field was aligned with a predominant hydrostatic stress field. As before, the results obtained showed a consistent pattern across the RVEs analyzed. Nevertheless, the strain at which the first damage events occurred was different for each RVE. This is due to the fact that different fiber strength values were obtained for each RVE. By comparing the separate effects of matrix damage, interface damage (Figure 6, Figure 7 and Figure 8), and fiber break (Figure 9 and Figure 10), it is clear that each damage mechanism induced a characteristic reorientation of the octahedral stress field marked with a unique combination of $\left|\lambda_{1}\right|$, $\left|\lambda_{2}\right|$, and *θ*_1_ values. This PCA analysis provides a new valuable numerical insight into the complex damage behavior of UD-FRP that cannot be obtained by conventional mechanical analysis.

#### 3.2.5. Modified PCA of the Longitudinal Tensile Strain Simulations (Matrix/Interface Damage and Fiber Breaks)

**Figure 11:**
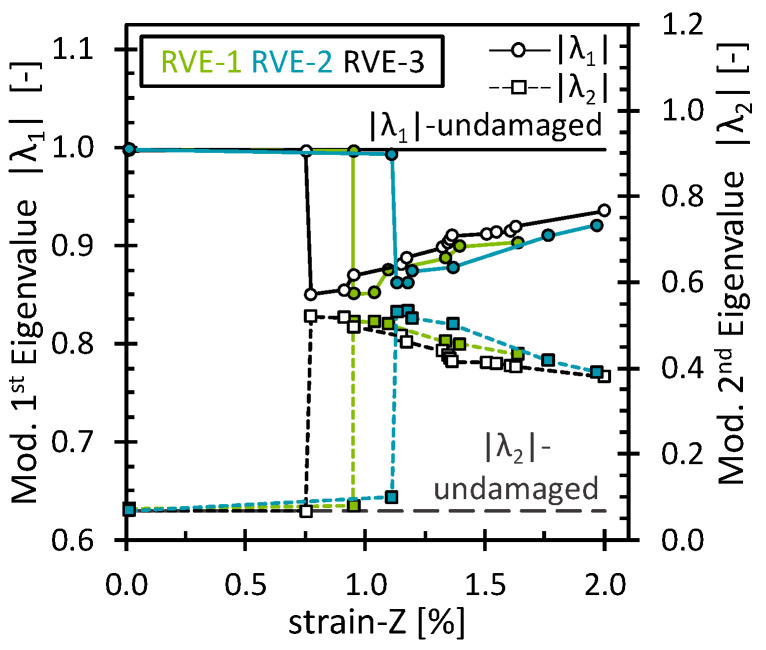
Modified eigenvalues for combined damage as a function of the longitudinal strain.

**Figure 12:**
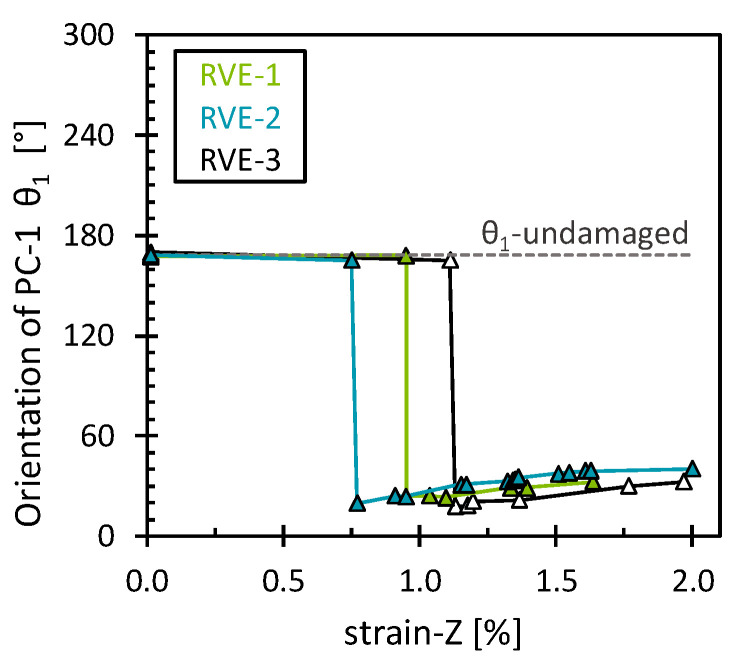
Orientation of the first eigenvector for combined damage and the interface damage as a function of the longitudinal strain.

The effect of the matrix damage, interface damage, and fiber breaks on the octahedral stress field was analyzed for simulations under the 2% longitudinal tensile strain. As observed before, the overall stiffness of the RVE was drastically affected as more fibers were damaged. Furthermore, the load-bearing capacity of the matrix was also compromised as matrix damage increased during the simulation. The stress–strain curve and the evolution of the octahedral stress can be found in Appendix C (Figure A7). Interestingly, by including a matrix damage model, two out of three simulations met the convergence conditions and reached the final strain of 2%, even though a higher degree of damage was achieved. This suggests that matrix damage mitigated large stress discontinuities. Figure 11 shows the evolution of $\left|\lambda_{1}\right|$ and $\left|\lambda_{2}\right|$ through the deformation process. As expected, both eigenvalues remained unchanged as long as no damage events occurred. In general, the behavior of the eigenvalues was very similar to the analysis without matrix damage (fiber breaks only). An initial abrupt change in the modified eigenvalues was observed as soon as the first break occurred, at an average longitudinal strain of 0.98 ± 0.12%. Significant changes in the modified eigenvalues were observed with each subsequent new fiber break. Thus, with the first fiber break event, the octahedral stress field became less oriented toward PC-1 and more oriented toward PC-2. Furthermore, the strain to fiber break value obtained was in statistical agreement with the probability of failure as reported by Andersons et al. [44]. This indicates a redistribution of the stress field, forming a small cluster of high octahedral stresses, presumably corresponding to the elements around the fiber break. Furthermore, with each subsequent fiber break, the eigenvalues reversed their behavior, suggesting that the octahedral stresses tend to maintain the pattern established by the initial break, with each subsequent fiber break generating stress states that conform to this initial pattern. However, in contrast to the previous analysis, it can be observed that some matrix damage accumulated prior to the first break. This was more evident for the RVE-2 in Figure 11 where $\left|\lambda_{2}\right|$ deviated slightly from its undamaged value. Furthermore, no steady state of $\left|\lambda_{1}\right|$ and $\left|\lambda_{2}\right|$ was observed. In this case, however, a gradual change was observed during the period between fiber break events, which is attributed to the matrix and interface damage’s progressive nature. Figure 12 shows the orientation of the first eigenvector (*θ*_1_). Here, the stress field rotated clockwise immediately after the first fiber break, with a gradual rotation thereafter. For *θ*_1_, a mean value of 29.28° ± 6.76° was obtained after the first break, suggesting that although a small gradual rotation of the eigenvector was observed, the stress field aligned with a predominant hydrostatic stress field. Furthermore, the rotation of the first eigenvector, attributed to the progressive nature of damage in the matrix and interface, indicated a slight shift of the stress field towards a pure shear configuration. This suggests that both matrix and interface damage contributed to the shear redistribution of the stress field.

Additionally, it is observed that drastic interface damage followed after a fiber break event, but since no full damage state of numerous interfaces was reached, no clear stagnation effects were able to be observed as in the previous analysis. As before, the results obtained showed a consistent pattern across the RVEs analyzed. Still, the strain at which the first break events occurred was different for each RVE. This was due to the fact that different fiber strength distributions were obtained for each RVE. Nevertheless, it was evident that *θ*_1_ indicated whether the stress field was more or less oriented towards a hydrostatic or octahedral shear stress field, while the combination of $\left|\lambda_{1}\right|$ and $\left|\lambda_{2}\right|$ indicated the degree of dispersion of the stress field. By comparing the effects of matrix damage, interface damage, and fiber break, it is clear that each damage mechanism induced a characteristic redistribution of the octahedral stress field. Contrary to the analysis with the isolated damage mechanism, it was not possible to distinguish a unique combination of $\left|\lambda_{1}\right|$, $\left|\lambda_{2}\right|$, and *θ*_1_ values that allow differentiation of each damage mechanism. However, it can be observed that the matrix and interface damage produced a shear redistribution of the matrix stress field, while the initial fiber break established the general form of the octahedral stress distribution. Furthermore, $\left|\lambda_{1}\right|$ and $\left|\lambda_{2}\right|$ indicated the degree of dispersion of the stress field. Further numerical analysis should be carried out to extend the analysis and interpretation of the principal components. Nevertheless, the results provide valuable numerical insight that cannot be obtained by conventional mechanical analysis.

## 4. Conclusions

In this paper, principal component analysis (PCA) was implemented to analyze the matrix stress field of undamaged and damaged RVEs. For this, the stress field was divided into its octahedral stress components. It was observed that traditional eigenvalues depended on the mesh of the RVE, since they varied proportionally to the number of integration points of the RVE. A modification of the PCA is presented to obtain mesh-independent eigenvalues, allowing for a reliable comparison of the internal stress distribution between different RVE structures. It was found that these modified eigenvalues and the orientation of the eigenvectors provided valuable insights regarding damage initiation and evolution within the RVEs. Thus, the orientation of the first principal component, denoted as *θ*_1_, serves as an indicator of the predominant stress state composition within the octahedral stress field, where a *θ*_1_ value of 0° or 180° indicates a predominant hydrostatic stress state and a *θ*_1_ value of 90° or 270° corresponds to a predominant shear stress state. Furthermore, the magnitude of the modified eigenvectors indicates the degree of dispersion of the stress field. Furthermore, the PCA results suggest that the accumulation of matrix damage became significant for the composite’s stiffness at a strain of 1%, while fiber breakage began at an average longitudinal strain of 0.98 ± 0.12%. This analysis reveals that each damage mechanism left a distinct signature in the redistribution of the matrix stress field, i.e., a unique fingerprint, characterized by a unique combination of the principal component values. This provides a new and valuable numerical insight into the complex micro-damage behavior that cannot be obtained by conventional mechanical analysis. The results obtained show that PCA enhanced the interpretability of the complex internal stress distribution and changes within the microstructure of an FRP by reducing its dimensionality. Although consistent results and trends in the principal component values were obtained, further analysis is required to correlate those values with additional damage mechanisms and loading conditions. In addition, the presented PCA approach is expected to be implemented in future research to investigate fatigue-induced micromechanical damage under cyclic loading conditions, as well as the effects on the matrix residual stresses due to temperature variations.

## Figures and Tables

**Table 1:** Mechanical properties of the fiber and matrix.

Mechanical Properties		Toray T700 Carbon Fibers [29]	LY556/H917 Epoxy Matrix [30]
Longitudinal modulus [GPa]	*E* *_∥_*	230	3.15
Transverse modulus [GPa]	*E* *_⊥_*	15	-
Longitudinal shear modulus [GPa]	*G* *_⊥∥_*	15	1.17
Transverse shear modulus [GPa]	*G* *_⊥⊥_*	7	-
Longitudinal Poisson’s ration [–]	*ν* *_⊥∥_*	0.2	0.35
Transverse Poisson’s ration [–]	*ν* *_⊥⊥_*	0.5	-
Tensile strength [MPa]	*σ_f_*	4900	93

*∥*—parallel to the fiber direction, *⊥*—transverse to the fiber direction.

**Table 2:** Parameters for the stiffness degradation model.

Model Parameter		Matrix	Interface
*A*	[–]	1.0^−6^	5.0^−19^
*n*	[–]	10.0	8.0
*b*	[–]	0.5	0.5
$a_{I}$	[–]	75.0	55.0
$a_{II}$	[–]	10.0	10.0
$D_{I}$	[–]	0.1	0.25
$D_{II}$	[–]	0.5	0.75

**Table 3:** Weibull parameters [21,40].

*L* [µm]	*L*_0_ [mm]	*m* [−]	$\sigma_{0}$ [MPa]	*γ* [−]
2.0	30.0	3.38	4170	1.0

**Table 4:** PCA results for the RVE with regular square fiber arrangement.

RVE Size		Longitudinal Strain 2%					Transverse Strain 2%				
Fibers	Integration Points	*λ*_1_	*λ*_2_	|*λ*_1_|	|*λ*_2_|	*θ*_1_ [°]	*λ*_1_	*λ*_2_	|*λ*_1_|	|*λ*_2_|	*θ*_1_ [°]
1	2305	1.019	0.345	0.993	0.116	161.2	0.937	0.419	0.913	0.408	30.3
4	9220	1.441	0.489	0.993	0.116	161.2	1.325	0.592	0.913	0.408	30.3
9	20,745	1.765	0.599	0.993	0.116	161.2	1.622	0.725	0.913	0.408	30.3
25	57,625	2.278	0.773	0.993	0.116	161.2	2.094	0.936	0.913	0.408	30.3

## Data Availability

The original contributions presented in the study are included in the article. Further inquiries can be directed to the corresponding author.

## Appendix A

Microscopy analysis and details of the PCA mesh independency analysis.

## Appendix B

Damage evolution and octahedral stress field for the 2% transverse strain simulations.

## Appendix C

Damage evolution and octahedral stress field for the 2% longitudinal strain simulations.
